# The role of different SIRT1-mediated signaling pathways in toxic injury

**DOI:** 10.1186/s11658-019-0158-9

**Published:** 2019-05-30

**Authors:** Zhihua Ren, Hongyi He, Zhicai Zuo, Zhiwen Xu, Zhanyong Wei, Junliang Deng

**Affiliations:** 1 Key Laboratory of Animal Disease and Human Health of Sichuan Province, College of Veterinary Medicine, Sichuan Agricultural University, Chengdu, 611130 Sichuan Province China; 2 The College of Animal Science and Veterinary Medicine, Henan Agricultural University, Zhengzhou, 450002 Henan Province China

**Keywords:** SIRT1, Toxicant, SIRT1 activator, Signaling pathway

## Abstract

Common environmental pollutants and drugs encountered in everyday life can cause toxic damage to the body through oxidative stress, inflammatory stimulation, induction of apoptosis, and inhibition of energy metabolism. Silent information regulator 1 (SIRT1), a nicotinamide adenine dinucleotide-dependent deacetylase, is a member of the evolutionarily highly conserved Sir2 (silent information regulator 2) superprotein family, which is located in the nucleus and cytoplasm. It can deacetylate protein substrates in various signal transduction pathways to regulate gene expression, cell apoptosis and senescence, participate in the process of neuroprotection, energy metabolism, inflammation and the oxidative stress response in living organisms, and plays an important role in toxic damage caused by toxicants and in the process of SIRT1 activator/inhibitor antagonized toxic damage. This review summarizes the role that SIRT1 plays in toxic damage caused by toxicants via its interactions with protein substrates in certain signaling pathways.

## Introduction

Silent information regulator 1 (SIRT1) is a histone deacetylase of nicotinamide adenine dinucleotide (NAD^+^), which mainly exists in the nucleus, and is a member of a family of well-studied mammalian sirtuins. SIRT1 interacts with protein substrates in a variety of signaling pathways (such as Wnt and Notch), participates in the regulation of most of the body’s physiological functions, and plays a central regulatory role in cell proliferation, differentiation, senescence, apoptosis, and metabolism, attracting attention from researchers in various disciplines [1, 2].

In our daily life, we are constantly exposed to various sources of chemical and physical injury in the form of drugs, environmental pollutants, ultraviolet radiation, and ionizing radiation. At high levels, toxic substances can cause toxic damage through a variety of mechanisms including oxidative stress, inflammatory stimulation, and inhibition of energy metabolism, causing serious harm to the body [3]. Studies have shown that SIRT1 can participate in toxic damage caused by toxic substances by interacting with protein substrates, such as the Forkhead-box transcription factor (FOXO) family, nuclear factor kappa B (NF-κB), peroxisome proliferator-activated receptor gamma-assisted activating factor-1 (PGC-1), and tumor suppressor p53 in some signaling pathways [4–8]. It also plays an antagonistic role under the activation of SIRT1 activator. Therefore, this review will focus on the interaction between SIRT1 and protein substrates in some signaling pathways to regulate the toxic damage process, providing a theoretical basis for further study of SIRT1.

### Pathway regulation of SIRT1 in toxicological damage

SIRT1 can catalyze the deacetylation of acetyl lysine of histone substrate and some non-histone substrates to regulate gene expression. It can participate in the regulation of apoptosis, the inflammatory response, oxidative stress, energy metabolism, and other processes by regulating different pathways [9, 10], playing an important role in toxicological damage.

### SIRT1/PGC-1α

PGC-1α is a transcription factor co-activator that affects most cell metabolic pathways. It influences mitochondria respiration, the reactive oxygen species defense system, and fatty acid metabolism by interacting with specific transcription factors [11–13]. Studies have shown that SIRT1 can enhance tissue antioxidant capacity by activating the transcription of PGC-1α and inducing the expression of superoxide dismutase (SOD) and glutathione peroxidase (GSH-PX) in cells [14–16]. Therefore, when toxic substances directly act on SIRT1 to reduce its expression, it can reduce the antioxidant capacity of tissues and cause oxidative damage to the body. Yuan [4] found that early lead exposure could reduce phosphorylated PGC-1α in the mouse cerebral cortex and SIRTl expression in the nucleus of cerebral cortex cells, increase the retention of PGC-1α in the cytoplasm, reduce the activity of GSH-PX and the GSH content, and reduce the antioxidant capacity. Excessive fluoride can also inhibit SIRT1, significantly downregulate the protein expression level of SIRT1, and cause central nervous system oxidative damage through the SIRT1/PGC-1α pathway [5]. SIRT1 can also regulate the function of PGC-1α in cells by regulating the acetylation and activity level of PGC-1α, as well as regulation of downstream transcription factors such as nuclear receptor peroxisome proliferator-activated receptor (PPAR), estrogen-related receptor (ERR), nuclear respiratory factor (NRFs), and mitochondrial transcription factor A (Tfam), further affecting mitochondrial production and function [17, 18], and regulating the metabolism of glucose and lipids [19]. In addition, structural damage or dysfunction of mitochondria also leads to the initiation of apoptosis, so SIRT1 can regulate the functional state of mitochondria and indirectly control apoptosis by regulating the acetylation level of PGC-1α. Regarding cadmium toxicity damage, studies have found that cadmium treatment can obviously inhibit the expression of SIRT1 and increase PGC-1α acetylation levels [8], damaging mitochondria and leading to mitochondrial dysfunction, and eventually inducing cell death processes such as apoptosis and necrosis [20–22], which may also be an important cause of hepatotoxicity induced by cadmium exposure. Valproic acid, an antiepileptic drug, also inhibits the protein expression level of SIRT1, causing hepatotoxicity [23] and mouse embryonic development abnormalities [24] through the SIRT1/PGC-1α pathway. Therefore, in the toxic damage caused by some poisons, the SIRT1-mediated PGC-1α pathway can play an important role by regulating the body’s antioxidant capacity and mitochondrial production and functional status.

### SIRT1/NF-κB

NF-κB is the master switch of the inflammatory response, which is usually connected to inhibitory protein inhibitor of NF-κB (IκB) in the form of a p65/p50 dimer. When stimulated, p65/p50 can be activated and transferred to the nucleus to regulate the transcription of various downstream inflammatory factors [25, 26]. The p65 subunit of NF-κB is the direct target of SIRT1, which, through deacetylation, can control the acetylation level of NF-κB p65 to regulate the transcription level of the downstream genes, including those encoding IL-1, tumor necrosis factor α (TNF-α), IL-8, IL-6, and other inflammatory factors [27–31], thus regulating the inflammatory response. In addition, NF-κB is also involved in the regulation of apoptosis [32], and SIRT1 regulates anti-apoptosis-related gene expression through NF-κB, such as inhibitor of apoptosis proteins (IAPs), the B-cell lymphoma-2 (Bcl-2) family, TNFR-associated factor (TRAF1, TRAF-2), JNK [33], etc., thereby controlling apoptosis. Regarding the toxic damage caused by fluorine, excessive fluoride can reduce the expression of SIRT1, so that NF-κB cannot be deacetylated, resulting in activation of the NF-κB signal, which causes neuronal apoptosis [34] and central nervous system damage [5, 35]. Studies have demonstrated that in an animal model of Alzheimer’s disease, the β-amyloid protein (Aβ) content in the brain is negatively correlated with the SIRT1 content in the same region [36, 37]. SIRT1 attenuates the neurotoxic effects of Aβ in Alzheimer’s disease by inhibiting NF-κB signaling in microglia [28]. The antitumor antibiotic doxorubicin has serious side effects linked with cardiotoxicity [38]. Xi and coworkers [39] found that intraperitoneal injection of doxorubicin can lead to increased expression of malondialdehyde (MDA) and NF-κB protein, and decreased expression of SOD activity and SIRTl, causing oxidative stress and inflammatory damage in C57BL/6 J mice. It can be seen from the above that SIRT1 can regulate apoptosis by controlling the level of deacetylation of NF-κB, thus affecting the toxic damage of some toxicants. However, the SIRT1/NF-κB pathway mainly participates in the toxic damage process of toxicants by the inflammatory response.

### SIRT1/FOXO

The FOXO protein family is widely involved in cell signal transduction, growth and development, apoptosis, and antioxidant stress, among which FoxO1 and FoxO3 are the most common. This family of proteins can activate or inhibit a variety of target genes, such as p27kip1 and cyclin D (CCND) CYR61, which regulate the cell cycle, the *bim* and *fasL* genes that mediate apoptosis [40], TNF and tumor necrosis factor-related apoptosis-inducing ligand (TRAIL) [41], and the RAD51 gene involved in DNA damage repair.

The complex interaction between SIRT1 and FOXO protects against oxidative stress [42–44]. On the one hand, SIRT1 upregulates the deacetylation of FOXO, enhances FOXO-induced cell cycle arrest, activates and promotes the FOXO/MnSOD pathway, increases the expression of manganese superoxide dismutase (MnSOD) and catalase (CAT) to resist oxidative stress, and promotes the repair of DNA damage during replication [45, 46]. On the other hand, after deacetylation of FOXO by SIRT1, FOXO can be degraded by ubiquitination, reducing the level of FOXO and inhibiting the ability of FOXO to induce cell death, thereby ultimately protecting cells from oxidative stress damage [47, 48]. In fluoride-induced central nervous system damage [5], doxorubicin-induced cardiotoxicity damage [49], and valproic acid-induced hepatotoxicity [23], the toxic effects on SIRT1 cause a decrease in expression, and a decrease in the level of FOXO deacetylation leads to an increase in apoptosis, leading to damage. Smoke from cigarettes has also been shown to cause oxidative stress damage in lung cells by acting on the SIRT1/FOXO pathway [50–52]. After activation of the SIRT1/FOXO pathway, the level of FOXO deacetylation not only regulates the oxidative stress of the body, but also involves the control of cell apoptosis and the cell cycle, which is a complex and interactive process. Therefore, the study on the role of this pathway in the toxic injury of related toxins should be more comprehensive and systematic.

### SIRT1/Nrf2

Nuclear factor E2-related factor 2 (Nrf2) is widely regarded as a transcription factor activated by oxidative stress that induces the coding of a series of antioxidant protective proteins and promotes the regulation of redox conditions in cells [53]. In addition, Nrf2 is also an important negative regulator of inflammatory cytokine activation and interleukin-1-mediated vascular inflammation [54, 55], and therefore participates in the process of inflammation. Some studies have shown that Nrf2 can be regulated by acetylation [56, 57], while SIRTI can activate Nrf2 transcriptional activity and upregulate Nrf2 downstream gene expression of genes such as those encoding SOD and GSH [58, 59]. Conversely, downregulation of SIRT1 expression significantly reduced Nrf2 protein expression [60]. Regarding the toxic damage caused by paraquat, some studies have found that overexpression of SIRT1 can deacetylate NRF2, increase the stability of Nrf2, promote the transport of Nrf2 to the nucleus, promote the transcriptional activity of Nrf2, enhance the resistance of cells to oxidative damage, and play a protective role in the AEC-II injury of mice caused by paraquat poisoning [61, 62]. It can be seen that the SIRT1/Nrf2 pathway can antagonize the oxidative damage caused by some toxicants by enhancing the antioxidant capacity of the body.

### SIRT1/p53

p53 can regulate the expression of a large number of downstream target genes, which in turn affects cell cycle organization, apoptosis, differentiation, and a number of other processes [63, 64]. SIRT1 enhances the expression of MnSOD by deacetylating p53, thereby increasing cellular antioxidant capacity [65, 66]. It is also negatively regulated by p53. When cells are under oxidative stress, SIRT1 can deacetylate the lysine residue at position 382 of the p53 protein and inhibit the activity of p53, thereby inhibiting the transcription of downstream target genes dependent on p53, such as CDKNIA and BAX, reducing cell apoptosis [67–69]. The p53-microRNA34a-SIRT1-p53 pathway has been shown to regulate the normal development and functioning of follicles [70]. In the process of reproductive damage caused by fluorosis, overexpression of SIRT1 can increase the levels of deacetylated p53, thereby antagonizing the reproductive damage caused by fluorosis and exerting a protective effect. In the toxic damage caused by some toxicants, SIRT1 can regulate the deacetylation level of p53, which can affect the antioxidant capacity of cells and regulate cell apoptosis. Gu et al. (2019) reported that SIRT1 plays an essential role in protection against fluoride-induced oxidative stress and mitochondria-dependent apoptosis in MC3T3-E1 cells. The SIRT1/p53/p21 pathway may be a potential therapeutic target for fluorosis [71].

**Fig. 1:**
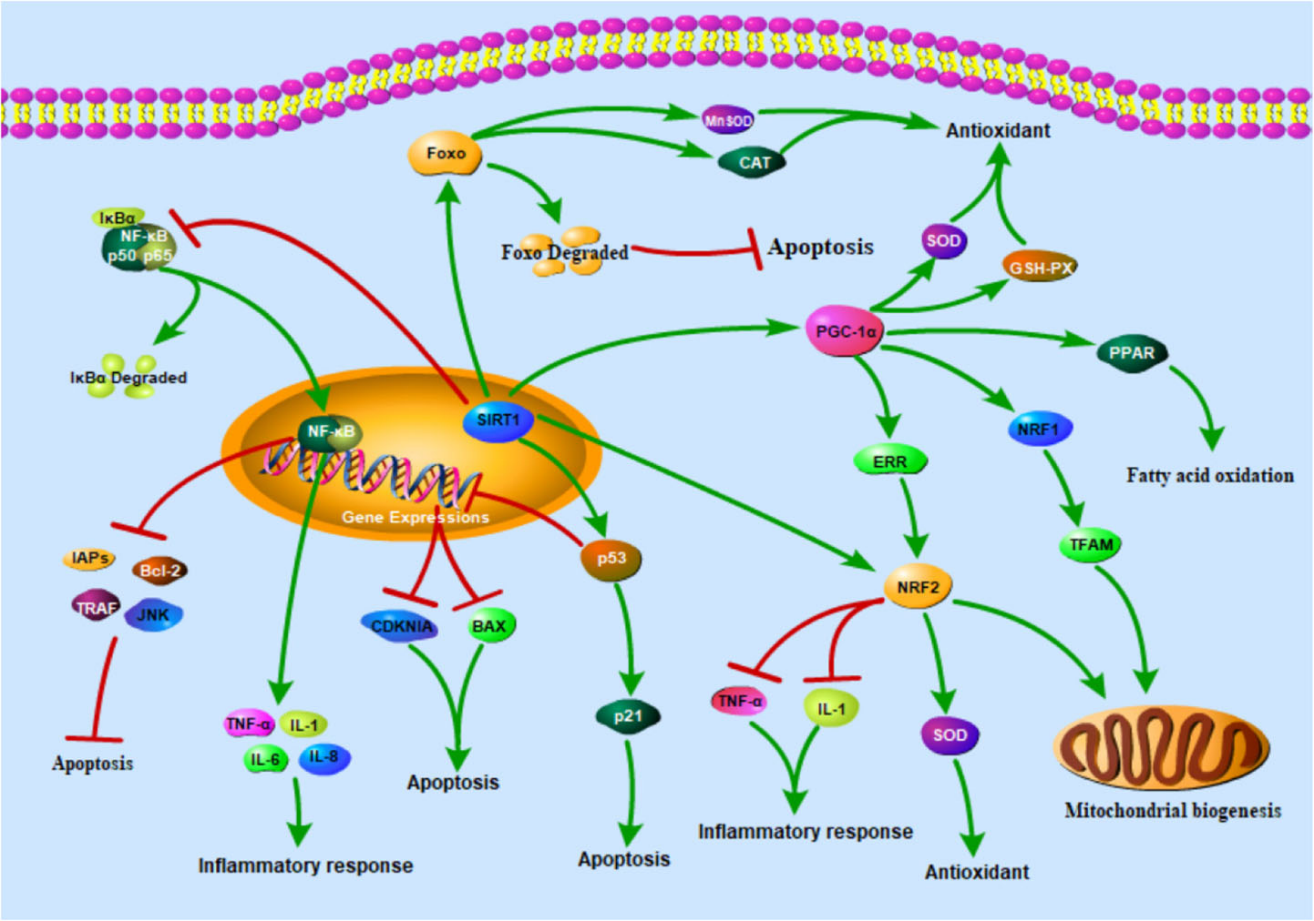
The role of different SIRT1-mediated signaling pathways

To sum up, the substrates of SIRT1 action and corresponding biological function are summarized in Table 1. Figure 1 showing the role of different SIRT1-mediated signaling pathways in toxic injury.

**Table 1:** Substrates of SIRT1 action and corresponding biological function

Substrate	Biological function
PGC-1α	Antioxidant stress, Regulate mitochondrial function and Regulating the metabolism of glucose and lipid
NF-ƙB	Inhibition of inflammation, Regulation of apoptosis
FOXO, p53	Control of cell cycle, Antioxidant stress and Regulation of apoptosis
NRF2	Antioxidant stress, Inhibition of inflammation

### The role of SIRT1 agonists/inhibitors in toxic damage caused by toxicants

To date, the most studied of the SIRT1 activators that antagonize toxic damage is resveratrol (Res). Res is a polyphenolic plant metabolite, and this family of metabolites were the first small molecule activators of SIRTl to be discovered [72]. Res is the most potent member of this family and can enhance the protein expression and activity of SIRTl [73, 74] and binds more easily to substrates following a change in the conformation of SIRTl [75, 76]. Res can upregulate SIRTl and inhibit the production of reactive oxygen species through the SIRTl/FOXO3 pathway to resist oxidative damage [77]. Res can also regulate heme oxygenase 1 (HO-1) expression through the Nrf2/ARE signaling pathway to protect PCI2 cells from oxidative stress damage [78]. Anekonda and colleagues [79] found that Res could reduce the intracellular calcium level, downregulate Bax expression, upregulate the activity of SIRTl and Ku70, and inhibit the activity of caspase-3 and cell apoptosis.

In lead-induced toxic injury, Res can increase the level of SIRTl to deacetylate PGC-1a, increase the content of PGC-1a, activate the function of PGC-1a as an NRF-1 co-activator, bind DNA with NRF-1, enhance transcription and activate oxidative phosphorylation reactions [80], thereby inhibiting the formation of Ap (1–40) in the cerebral cortex of lead-exposed mice, protecting mice against lead exposure-induced problems with spatial learning and memory [81]. In addition, Res can also activate SIRT1 and increase MnSOD resistance to lead oxidative stress damage through mitochondrial biogenesis [82]. It has been found that in amphotericin-induced acute lung injury, Res can reduce the level of injury through multiple pathways, including inhibiting apoptosis, anti-oxidation, and protecting endothelial cells, and can upregulate SIRTl and reduce the subsequent production of inflammatory cytokines [83]. In a study by Sang and coworkers [84], it was found that Res may reduce the expression of the proapoptotic gene *Bax* by increasing expression of the SIRT1 gene and the anti-apoptotic gene BCL2, thereby exerting an anti-apoptotic effect and reducing the toxicity of zearalenone.

In addition to Res, melatonin can improve the functional status of mitochondria by promoting their production via the MT1/SIRT1/PGC-1 signaling pathway, thereby protecting against the hepatotoxicity caused by cadmium exposure [8]. SRT1720, a SIRT1 specific activator, protected H_2_O_2_-induced senescent endothelium. It could protect against endothelial senescence and maintain cell function via the Akt/eNOS/VEGF axis [85]. SRT2104, also a SIRT1 specific activator, attenuated lipopolysaccharide-induced release of the cytokine interleukin-6 and inhibited activation of coagulation [86]. Rosuvastatin, a commonly used cardiovascular lipid-lowering drug, can upregulate the expression of SIRTl, further inhibiting the activity of NF-κB, terminating the release of downstream inflammatory mediators, and protecting against doxorubicin-induced myocardial toxicity. In addition, salvianolic acid B (SaLB) can also activate the overexpression of SIRT1 [48]. Overexpression of SIRT1 can phosphorylate FOXO3a and lead to overexpression of MnSOD protein. MnSOD is an important antioxidant protein and a major antioxidant enzyme in mitochondria. It is mainly used to scavenge reactive oxygen species [87]. Therefore, SaLB can also protect against the cardiotoxicity caused by doxorubicin.

Although it can be found from most current studies that SIRT1 activators mainly play an antagonistic role in toxic damage caused by toxicants, some studies have reported that SIRT1 activators can aggravate toxic damage caused by toxicants, while SIRT1 inhibitors antagonize toxic damage caused by toxicants. Cai et al. [88] found that toxicity of extracellular Zn^2+^ depended on entry, elevation in intracellular free Zn^2+^ ([Zn^2+^]i), a reduction in NAD^+^ and ATP levels, and dysfunction of glycolysis and cellular metabolism. While SIRT proteins are NAD^+^-catabolic protein deacetylases, Res and fisetin can potentiate NAD^+^ loss and Zn^2+^ neurotoxicity. In contrast, sirtinol, nicotinamide (NAM) and 2-hydroxynaphthaldehyde, inhibitors of the sirtuin pathway, attenuated both acute and chronic Zn^2+^ neurotoxicity. Lee et al. [89] found that NAM and sirtinol may alleviate high glucose/palmitate (HG/PA)-induced glucolipotoxicity to INS-1 beta cells by inhibiting the production of NAD^+^-depleting enzymes such as sirtuins. Moreover, NAM prevents NAD^+^ depletion and protects neurons against excitotoxicity and cerebral ischemia [90].

Finally, the main role of each SIRT1 agonist/inhibitor in toxic damage caused by toxicants are summarized in Table 2.

**Table 2:** The main role of each SIRT1 agonist/inhibitor in toxic damage caused by toxicants

SIRT1 modulators	Role in toxic damage caused by toxicants
Res	Antioxidant stress, Inhibits apoptosis, anti-inflammatory, protect endothelial cells, potentiated NAD^+^ loss
Melatonin	improve the functional status of mitochondria
SRT1720	Protect endothelial cells
SRT2104	Anti-inflammatory, inhibits activation of coagulation
Rosuvastatin	Anti-inflammatory
SaLB	Antioxidant stress
NAM	Prevents NAD^+^ Depletion
Sirtinol	Prevents NAD^+^ Depletion

## Conclusion

In summary, toxicant-induced injury affects SIRT1 expression, which then affects the expression and activity of downstream proteins, resulting in toxic damage. Upregulation of SIRT1 expression by SIRT1 activator can generally alleviate the toxicity of toxicants. SIRT1 can interact with proteins in various signal transduction pathways and regulate biological, physiological, and pathological processes. For example, SIRT1 can reduce the release of inflammatory factors by inhibiting the expression and activity of NF-κB in the NF-κB signal transduction pathway, thus alleviating the inflammatory damage caused by some toxicants. Therefore, an in-depth study of the role and mechanism of action of SIRT1 in toxic damage caused by poisons may provide new insight into therapeutic strategies to limit the toxic damage caused by poisons.

## Abbreviations

Aβ: β-amyloid protein
Bcl-2: B-cell lymphoma-2
CAT: Catalase
ERR: Estrogen-related receptor
GSH-PX: Glutathione peroxidase
HG/PA: High glucose/palmitate
HO-1: Heme oxygenase
IAPs: Inhibitor of apoptosis proteins
MDA: Malondialdehyde
MnSOD: Manganese superoxide dismutase
NAD: Nicotinamide adenine dinucleotide
NAM: Nicotinamid
NF-κB: Nuclear factor kappa B
Nrf2: Nuclear factor E2-related factor 2
NRFs: Nuclear respiratory factor
PGC-1: Peroxisome proliferator-activated receptor gamma-assisted activating factor-1
PPAR: Peroxisome proliferator-activated receptor
Res: Resveratrol
SaLB: Salvianolic acid B
SIRT1: Silent information regulator 1
SOD: Superoxide dismutase
Tfam: Mitochondrial transcription factors A
TNF-α: Tumor necrosis factor α
TRAIL: Tumor necrosis factor-related apoptosis-inducing ligand
